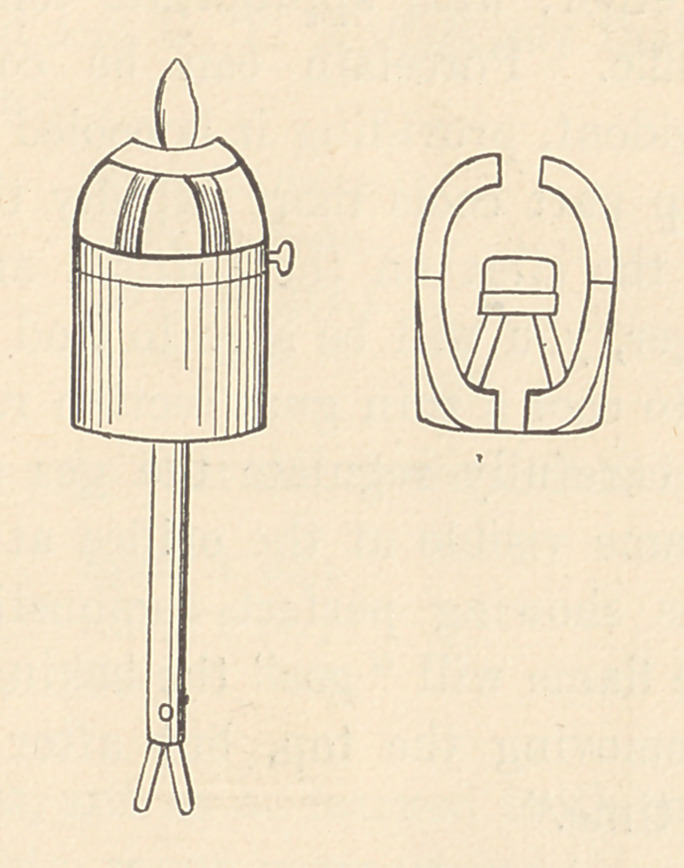# The Art of Carving and Baking Porcelain Teeth and Crowns

**Published:** 1904-01

**Authors:** R. T. Moffatt

**Affiliations:** Boston, Mass.


### International Dental Journal.

Vol. XXV. January, 1904. No. 1

### Original Communications.[1]

### THE ART OF CARVING AND BAKING PORCELAIN TEETH AND CROWNS.[2]

BY R. T. MOFFATT, D.M.D., BOSTON, MASS.

The carving of teeth by dentists as a part of their every-day practice is not at all common, and, in fact, I doubt if there are to-day more than fifteen or twenty men in the dental profession in the entire United States who really know anything about it. The carving of teeth, and the preparation of the bodies and enamels wherewith to do it, is so old that to the present generation it is entirely new. It is, in fact, nearly a lost art. This class of work is not taught in the dental schools, though to my mind it should be, if for no other reason than for the education of the eye and hand. Neither is it treated of in the text-books, except those published away back in the '50's. The reason for this lack of appreciation of a useful, practical, and really desirable art is somewhat difficult for me to explain. Those who have been fortunate enough

[1] The editor and publishers are not responsible for the views of authors of papers published in this department, nor for any claim to novelty, or otherwise, that may be made by them. No papers will be received for this department that have appeared in any other journal published in the country.

[2] Read before The New York Institute of Stomatology, October 6, 1903.

to acquire the art could not by any possibility do without it, unless sacrificing their artistic ideals of prosthetic work. It seems to me that the "commercial bug" has affected modern dentists to such a degree that in their desire to get rich quickly, they prefer methods that will enable them to turn out artificial teeth in quantity, rather than of a quality that would satisfy an artistic sense. There is as much difference beween a well-carved tooth and a "store" or moulded tooth as between an ordinary cheap photograph of a person and an oil portrait that is a speaking likeness of the subject.

Carving teeth is a delicate art, requiring skill, experience, and artistic taste. As any simple object needs skill and training in its imitation, the beginner must not expect to be able to produce the forms of natural teeth in porcelain without many failures and much perseverance. It will be necessary to study the shapes of many different types of teeth, those belonging to all the different temperaments, in order that the results for each case may not seem as if made from the same hand, or reproduced by means of a mould that is merely mechanically perfect. However, if one will persevere, more or less success may attend his efforts. It has been suggested that an art student, preferably a young lady, might be taught to carve and bake teeth for dentists with satisfaction to the latter and profit to herself. It is possible that this would work well, but it has not my approval, for this reason: for really artistic work that will imitate nature, I think none would be able to do it so well as a dentist, who, with practised eye and fingers, is constantly working over, examining, and repairing the natural teeth, and he best of all knows exactly what he wants as to color, form, and characteristics.

Porcelain teeth were first introduced to this country by a Frenchman, who brought some to Philadelphia about 1820. According to the "American System of Prosthetic Dentistry," Charles W. Peale, in 1822, and Samuel W. Stockton, in 1825, were the next after the Frenchman to manufacture porcelain teeth. They were soon followed by many others, and by 1838 mineral, porcelain, or incorruptible teeth, as they were variously called, had come into general use. Apparently those above mentioned did not carve teeth for individual cases, but they manufactured them from moulds so that they could make them in quantities.

In 1838 there appeared in the *Boston Annual Advertiser and Directory* the following advertisement. This was the only advertisement of the kind in the *Boston Directory* between 1825 and 1860.

IMPROVEMENT IN DENTAL SURGERY.

WHITE TEETH.

Removal to Granite Building, No. 14 Howard St.

Dr. B. T. Prescott, would inform his friends and the public generally, that he has removed from his old stand, Hanover, corner of Portland St. to the Granite Building, No. 14 Howard St., directly opposite Pemberton House (formerly Holland's Coffee House), where he continues to perform all the necessary operations on the teeth, both for their beauty and preservation. Particular attention paid to cleansing and filling carious teeth with gold, thereby arresting the progress of decay and rendering them useful for many years. Mineral teeth of superior quality inserted on the most reasonable terms.

Dr. Prescott, having obtained the latest and highly approved method of inserting the mineral or incorruptible teeth on gold plates, so recently introduced in Paris, feels assured that he cannot fail to give a general and acknowledged satisfaction to all who may favor him with their call.

Persons requiring operations on the teeth will please call and see specimens at his office, Stone Building, No. 14 Howard St., a few doors West of Concert Hall, Boston.

N.B.—Dentists supplied with mineral or incorruptible teeth, European and American, Wholesale and Retail, on the most reasonable terms. Orders from the country promptly answered.

As far as I have been able to determine, Boston seems to be unique in having fostered a taste for something better than manufactured teeth, turned out of moulds in quantities, entirely lacking in character and individuality. At the time of which I speak conditions in dentistry were much different from what they are to-day, as one would suppose, and these conditions should be understood before I proceed further with my paper. The principal usefulness of dentists at this time was in the extraction of teeth and the manufacture of artificial teeth, so that what we call laboratory work played a prominent part. Plates were then made entirely with metal and the teeth themselves were prepared in secret.

Among the most notable dentists of Boston in their time were the following, with date at which they commenced practice in Boston:

1826. W. P. Greenwood (a famous character), Nathan Cooley Keep (principal witness in Webster-Parkman murder case), Samuel O. Bemis, Thomas Barnes, Moses F. Randall, Richard R. Smith.

1828. Josiah F. Flagg, Thomas W. Parsons (afterwards the great Latin scholar).

1830. Daniel Harwood.

1836. Joshua Tucker.

1842. Elisha Tucker (brother to above).

1846. David Jordan, E. G. Leach.

1848. D. M. Parker, W. T. G. Morton (who needs no introduction).

1849. Willard W. and Benjamin Codman (the latter afterwards founding the firm of Codman & Shurtleff), A. F. Preston.

1851. Jacob L. Williams.

1853. E. T. Wilson.

1855. I. A. Salmon, A. L. Snow (inventors of an automatic mallet), H. D. Osgood (who was constantly in practice until his death last August).

1856. C. E. Dearborn (who is still alive, aged eighty-three).

1857. George T. Moffatt (afterwards Professor of Operative Dentistry, Harvard Dental School).

1858. I. J. Wetherbee (afterwards Dean of Boston Dental School).

1859. Henry Jordan.

1860. Samuel F. Ham, W. E. Woodman.

1862. Thomas B. Hitchcock.

1864. Thomas H. Chandler (afterwards Dean of Harvard Dental School), Nathaniel W. Hawes (afterwards Adjunct Professor of Operative Dentistry, Harvard Dental School), Aaron H. Parker.

1865. Thomas Cogswell, E. N. Harris.

1867. John T. Codman.

1868. R. R. Andrews, Peter Burchaell, L. D. Shepard, John T. Stetson, F. F. Gage.

1870. Charles S. Bartlett.

1872. E. P. Bradbury, D. M. Clapp, D. D. Dickinson, T. O. Loveland.

Out of the above, those whom I have found to have been carvers of teeth were the following: N. C. Keep, Daniel Harwood, Joshua

Tucker, C. E. Dearborn, W. E. Woodman, George T. Moffatt, Henry Jordan, Thomas H. Chandler, S. F. Ham, Aaron H. Parker, Peter Burchaell, F. F. Gage, and Charles S. Bartlett, truly not a large number for a period of forty-six years, from 1826 to 1872.

Nathan Cooley Keep was probably the first carver of teeth in Boston. His work was celebrated for its artistic merit and its individuality. It was by his identifying some teeth which he had carved, and which fitted some models that he had saved, that Professor Parkman was hanged for the murder of Dr. Webster. The porcelain of which Dr. Keep made his bodies and enamels was considered very beautiful, and was noted for the lifelike appearance that it gave his teeth. In those days knowledge in dentistry was kept secret by those who possessed any, and consequently posterity has no authentic formula of Dr. Keep's, as it was said that he kept them in his head, and his secrets died with him.

Next to Dr. Keep was Daniel Harwood. It was related of him that in 1838 he went to Philadelphia, and finding a broken saucer that seemed to be made of material suitable for artificial teeth, he got the receipt for its composition and then constructed some porcelain for incorruptible teeth. He was an artistic carver of teeth and a very busy man. I was told recently by Dr. A. H. Parker that Dr. Harwood had told the latter that many a time he (Harwood) had sat up nearly all night carving teeth, while his wife held a lamp for him to see by. In these days under discussion it was the custom for a dentist with a large practice to take under his training and tuition some promising young man, treating him like an apprentice. These apprentices would pay one thousand dollars for the privilege of being with a man like Dr. Harwood. Dr. Harwood had in all sixteen of these young men or apprentices, four of whom were David M. Parker, C. E. Dearborn, A. H. Parker, and Wilbur Parker, the two latter being brothers. David Parker never carved teeth, but the others did. I commenced my dental career under Dr. A. H. Parker, entering as a laboratory boy, at the age of sixteen, and after four years with him, entered the Harvard Dental School. After graduation I was associated with him for two years. I commenced to carve teeth before I really had studied dentistry, and while with Dr. Parker I had the valuable experience of running the old-fashioned coal and coke furnaces, helping in the manufacture of the improved gas furnaces, grinding and preparing titanium, silex, felspar, and clay, mixing the bodies, enamels, etc. I afterwards received some valuable information from my father on these same subjects and as he studied with Dr. Joshua Tucker, and A. H. Parker studied with Dr. Daniel Harwood, and Drs. Harwood and Tucker were at one time associated, I feel that I am rightfully descended, by the "carving genealogical tree," from the pioneer workers in porcelain dental art.

C. E. Dearborn's teeth had a sameness of expression that suggested a mechanical treatment. W. E. Woodman, I have understood, carved beautiful and artistic teeth. He left a son, who is now the tooth-carver for the Boston Dental Laboratory. Of the work of Henry Jordan, T. H. Chandler, and S. F. Ham I can say little, as I have never seen the work of either of them. I will give you later, however, some formulas of Dr. Chandler's and Dr. Ham's.

Porcelain for carving teeth is an entirely different material in every way from the material at present on the market that is used for porcelain inlays and restorations. Porcelain teeth are composed of two parts, the body representing the dentine of natural teeth, and the enamel representing what its name implies. The body is made of silex (pure quartz), felspar, and kaolin, or clay, and an oxide of a metal for the coloring. Enamel is made of felspar and a small proportion of silex, and the coloring pigment.

The qualities of a good body are a natural, "bony, lifelike appearance, density sufficient to withstand the strains of mastication, power to withstand the heat of the blow-pipe, a not undue amount of shrinkage during baking, and sufficient plasticity when unbaked to permit of easy handling during carving. The enamel should melt at a temperature slightly below that of the body, for if too fusible it will melt into, rather than upon the latter. Porcelain undergoes a notable diminution of volume during baking, varying from one-eighth to one-fifth according to the formula and amount of moisture, but the same mixture, if moistened always to the same consistency, will contract an amount that is always constant, and which can be properly allowed for. The density is increased with the contraction.

Bodies are best kept in a moist state, as they undergo a process of mouldering, owing to a fermentation of organic matter. This process seems to make the bodies smoother, more homogeneous, and more plastic. It is said that the Chinese have handed down the moistened clay, used for their porcelain wares, through their families for several generations before it is used for its ultimate purpose. If kept too wet the suspended particles will settle into a cake at the bottom of the vessel and the water rise to the top. This settling can be overcome by the addition of acetic acid, which increases the density of the water, but it has the disadvantage of making a rather nauseating odor.

As a consideration of the physical qualities of the constituents of bodies and enamels may not be amiss, I will briefly mention them.

Silex, or quartz, is the main constituent of body, as it gives density and stability and maintains the shape of the tooth during the fusing process; it is practically infusible. It varies much in quality, that from some localities being better than that from others, most of the best coming from Pennsylvania. As the method for reducing silex to a degree of fineness suitable for the purpose under consideration is well described in the "American Text-Book of Prosthetic Dentistry," pages 226, 227, I will omit it here and refer you to that. Coarse silex is used on the planche or tray as a bed for the teeth and to prevent the fusion of the latter to the tray.

Felspar, when finely ground, is a white powder that looks so much like finely ground silex that care must be used not to get them mixed until you are prepared to do so. Felspar, commonly spoken of as spar, is the flux which binds the particles of silex and clay together in the baked tooth. It is the main constituent of the enamel, and it gives the glaze to the tooth.

Kaolin, or clay, should be of the very finest, free from sand and mica or any traces of iron. I have seen blue clay and yellow clay of vastly different appearance in large lumps, but when properly prepared and mixed in the body there was no appreciable difference. Clay gives plasticity to body and assists in making an opaque, bony look. All clays are not plastic, however, nor are they always pure. When perfectly pure, clay is infusible even at the highest heats, but it will soften and become lustrous.

The coloring pigments are oxides of various metals, those of titanium, cobalt, chromium, and nickel being most used, and giving respectively the colors yellow, blue, green, and dark brown. Platinum "sponge" gives a grayish-blue color, and purple of Cassius, a preparation of gold, is used to give red and pink tints for the gum. The preparation of this pigment is a matter of great nicety, and sometimes the most experienced fail to get the desired results. The oxide of titanium can be made to give many shades of yellow, from a good warm orange-color to a pale canary yellow, according to its purity, the fineness to which it is ground, and to the presence or absence of iron. If coarsely ground a warm orange yellow will obtain, while fine grinding will give a lemon yellow. The presence of iron will make it look muddy and brownish. Some titanium containing iron, which I use, will give the black necessary to imitate tobacco stains. The art of tinting porcelains depends upon a knowledge of the management of the vitrifiable pigments. Like all other pigments they may be so mixed as to produce a great variety of tone and tints, but, unlike common coloring matters, chemical changes and reactions take place among them at the high heat to which they are necessarily exposed, so that this latter factor should also be taken into account in the preparation and use of the colors.

Frit is a mixture of a metallic oxide and felspar. By this means a greater subdivision of the pigment is secured, and hence a more thorough permeation of the mass to be colored. Dr. Parker does not frit his titanium, but he does his chromium and the other oxides.

Floating is a process of preparing porcelain materials so as to do away with much of the laborious grinding. The silex, spar, clay, or titanium is mixed with a large quantity of water, stirred, and the heavier particles allowed to settle from one-quarter to one-half hour. The water containing the suspended material is then drawn off and allowed to settle from twenty-four to forty-eight hours, when the water is again drawn off and the material at the bottom dried, and it is then ready for use. If allowed to settle too long, the material will be too fine to be of use for tooth body or enamel. Some felspar that Dr. Parker allowed to settle for four days was too fine, and was useless on account of excessive shrinkage.

We now come to the practical manipulation of the body and enamel in order to make a tooth. First, the instruments. These are few and can be made by any dentist. A "carving-knife," with a small blade about an inch long, three-sixteenths of an inch wide at its widest part, and about 26 gauge; it should have a wooden handle to secure lightness, and be about the size of an ordinary lead-pencil and four inches long. A similar knife with a very thin blade, say 36 gauge, and only about five-eighths of an inch long and one-fourth of an inch wide for separating the teeth. Formerly a bow of whalebone with a fine cotton thread was used for this purpose, but it is not so good or so handy. A little drill for making holes for the pins. A small, flat separating file. A small pair of tweezers with the points grooved for holding a pin, and having a snap catch, so that you can hold a tooth by the pins while enamelling it. A few small camel's-hair brushes for applying the enamel. In addition, you should have handy a small dish or cup full of *clean* water, a little bottle of olive oil with a brush, and a spirit lamp, or a small Bunsen burner with a clean flame. The blade of the carving-knife should be highly polished and the edges dulled and smooth rather than sharp.

The actual carving of a single tooth is a very easy matter, provided one is skilled with his fingers and has an artistic taste. As the description of the carving of a single crown will illustrate the method as well as for a whole set, I will take the simplest form which comes to us, and endeavor to make plain the making of a crown for an upper central incisor. We will suppose that the natural crown of an upper right central incisor has been broken off, leaving a good solid root, and that the other front teeth are all present and in good shape. After preparing the end of the root as you consider proper (and here let me state that you can give it almost any shape you wish, leaving the end practically as you find it, with the exception of removing decay), you fit your platinum post in the canal, giving it whatever direction you desire. Now take a plaster impression of the pin in the root, the end of the root, and at least two teeth on either side of the space to be supplied with the artificial substitute. Any other kind of impression material will not do; plaster *must* be used if you would have an artistic and accurate result. If you are to make a lateral, you should be sure to get an accurate impression of the other lateral, in order that you may have a guide that will enable you to imitate any natural characteristic peculiar to that particular patient's teeth. The same thing applies to cuspid and bicuspid teeth. Make sure that your impression is correct and that the pin has come away in the right place. If the impression breaks, fit the pieces together and wax them so that they will not fall off; then shellac and oil the impression, taking care not to use too much oil; then melt a very thin film of yellow wax on the part of the pin that projects from the impression and that was up in the root-canal.

Now plaster into your impression, and when sufficiently hard cut away the impression carefully, and you should have an accurate model of the patient's teeth, with the end of the root and the pin in place exactly as in the mouth. As soon as you have taken the impression of the teeth, after allowing the patient to rinse the mouth of stray bits of plaster, you should seal the root-canal with temporary gutta-percha. Now take a bit of yellow wax about the size of a walnut and warm and soften it, then press it into the space of the lost crown, and, pressing it firmly into place, let the excess of wax take an impression of the adjoining teeth and then let the patient bite into it, keeping the teeth closed for about two minutes, or until the wax hardens. Carefully remove the wax, taking great care not to distort it, and you will have an accurate occlusion of the lower teeth. Now, with your sample colors select the correct shade, and you can dismiss the patient. All the above should not take more than forty-five to fifty minutes. Now take your model with the pin still in it, and, turning it over in your hand so that the bottom or reverse side presents, make a hole of half an inch diameter in the direction of the apical end of the pin. As soon as you come upon the end of the pin, stop. Now take a fine pointed instrument and gently push the pin out from the impression, if necessary, first gently warming the pin to soften the film of wax before mentioned. Now properly shellac and oil the model, first making grooves or depressions that will guide the "bite half" always to its proper position; then take your little wax impression and bite and set it in its proper place upon the model. Now cast the bite in plaster, and when the latter is hard enough soften the wax in warm water and separate.

You now have an accurate and correct working model. Shellac the bite half and the entire model outside, where your hands or the tooth body would come in contact with it, the object of this being to keep the plaster from getting into the body and to show any scars or marks made upon the model. Next oil the model with olive oil at any part where the body will be placed. Now take an amount of the body sufficient to fill the space, and have it moistened with clean water to the consistency of putty; mix it in the palm of your hand with an ivory, bone, or celluloid spatula (the flat handle of an old tooth-brush will do nicely) until it is entirely free from air bubbles. Then put your pin back in place on the model, pack the body around it, and fill in the entire space, then close the bite half of the model. Next, pat it gently in with the finger in order to make the body as dense and solid as possible; it is even better to squeeze it into position. Now, with the carving-knife you can trim off the surplus body and gradually work it into the shape desired; you can cut, or scrape, or model with the flat of the knife as seems to be required. If you cut off too much, dip the knife-blade in the clean water and touch a drop to the body, then add a little more to give the contour desired; you will find there will be no difficulty in this patching. The tooth should be carved about one-fifth larger than you wished the finished tooth to be, to allow for shrinkage. Sometimes a little smoothing with a moistened camel's-hair brush will make the tooth look more as you wish. If this were a tooth for a plate instead of a crown that we are carving, you could put the platinum pins in now, using head or looped pins for rubber work or headless pins for gold plate work. Dip your drill in water, drill your hole wherever you desire the pin to be, then dip the pin in water, carry it to place, and insert in the hole, and then pat the body down around it to make it solid. Care must be used not to confine an air-bubble at the end of the pin, or you will weaken the tooth, or have a miniature balloon when the tooth is baked. Gently dry the tooth over a spirit lamp and remove from the model. Place some clean coarse silex on your tray or planche and lay the tooth upon this. Dry it out slowly over a Bunsen burner and then bake to a biscuit bake. Partially baking a tooth so that it has about the consistency of a piece of chalk is called biscuiting, or cruzing. The object is to make the tooth more easy to handle, and this final shaping should now be given the tooth. This is also the best time, in my opinion, to put the pins in, although it can be done when the body is soft. In the Parker gas furnace the biscuiting takes three minutes; in the Hammond No. 2 Electric, if you start with a cold furnace you can do the drying out in ten minutes on the first notch, and then heating up for one minute on each consecutive notch, give it four minutes on the sixth notch and then shut off current and cool down. On a biscuit bake you can take the cruzed tooth out at once without danger of cracking it. The biscuited tooth can now be handled with comparatively little danger of injury, and any smoothing up or finishing touches can be given now. Some prefer to put in the pins at this time. Burs in the engine can be used to drill the holes for the pins, and if in making a crown you desire one with a hole through it for the post, in preference to baking the post in, you can put it in now. Sand-paper disks in the engine can be used to cut or trim the tooth should you have cruzed it too hard.

### ENAMELLING.

After the tooth has been biscuited it is ready for enamelling. The tooth or crown should be held with tweezers, having a catch which will lock it firmly. The tooth should be held in a horizontal position, with the cutting edge towards you. The enamel should be in a small dish or saucer, such as is used by artists for water-color paints, and conveniently placed within reach. The enamel should be moistened with clean water to the consistency of thick cream. The enamel is applied with a small camel's-hair brush, the brush sopping up sufficient enamel to have it flow readily from the point of the brush on touching the tooth. It is better to have the tooth slightly warm, as the moment the enamel touches the tooth it is absorbed and dried at once upon its surface. The enamelling should commence at the cutting edge of the tooth. A ridge of the enamel should then be made along the cutting edge of the tooth, then from this a line of enamel should be made upon the centre of the tooth, parallel with its long axis, so that it makes a guiding line to show to what thickness the enamel should be applied over the remaining surface of the crown. The enamel should be applied in just the same manner as the natural enamel is found upon the tooth; in other words, quite thick on the cutting edge and tapering off to nothing at the neck. Care should be used to lay the enamel on without bubbles, or without getting frothiness. After the crown surface is enamelled about as you think it should be, little strokes with a moist brush, or with a small carving-knife, will enable you to put on artistic touches or characteristics which you wish to bring out in the finished tooth. If you wish to stain the tooth to imitate a tobacco stain or green stain, now is the time to apply it. This stain is applied on top of the enamel previously put on. When this is accomplished to your satisfaction, the tooth should be dried in the flame of a spirit-lamp. The tooth is now ready for baking.

Dr. Parker's directions for enamelling are to "wet the enamel with water to the consistency of thin cream, and lay or paint it on with a camel's-hair brush, in thickness and contour exactly like the natural enamel. A good effect can be produced, if desired, by too heavily enamelling a tooth, then, when baked, grinding off to match, and polishing in lathe with a buff-wheel or reglazing the surface in the furnace. In this way a greasy, dull surface is made, which has a more natural appearance than the highly glazed, crockery-like surface which is so much the characteristic of artificial teeth. If the color is not deep enough, a second coating of enamel can be brushed on and the tooth rebaked. A second bake always deepens the color, and makes the tooth more transparent. Another desirable effect is secured by levelling the enamel surface with a carving-knife after it has been laid on with the brush and while it is still wet and plastic; characteristic elevations and depressions can now be made. To a beginner a good, practical view of the enamel can be had from a section of a superior central incisor. Gum enamel is applied with a carving-knife."

FURNACES.

The early and common form of furnace was made of fire clay, bound and secured with wrought iron bands and hoops. (See illustration.) It was cylindrical in shape and made in three parts. The total height was about three and a half feet, and the diameter eighteen inches. The lower part contained the grate and ash-pit, the middle section contained a fire-clay muffle placed horizontally with external communication by a door, and the dome-shaped top section had two openings, one directly on top, in the centre, for the smoke-pipe, the other on the front side for the introduction of the fuel, usually coal. The muffle had to be securely luted so as to make it impervious to the gases from the coal, which would have "gassed" and spoiled the work. The external opening to the muffle was closed by a fire-clay door, which was perforated with a hole of about an inch diameter. Through this hole was arranged a clay stopper carrying a platinum wire, on the free end of which was placed a small quantity of body and enamel with each baking, so that the progress of the fusion could be determined from time to time. These furnaces made a lot of dirt. The time of baking varied according to the draft, but it was practically an all-day piece of work, including the gradual cooling off, and the muffles would sag and sometimes break and drop the work into the fire.

About 1870 Dr. A. H. Parker, of Boston, constructed a furnace on this principle, but of much smaller dimensions, it being about the size of a man's silk hat. In this furnace he used coke, which gave a fire more easily managed, and which reduced the dirt, time, and labor to about one-half. In 1889 he invented what came to be known as the Parker Improved Gas Furnace, a little cylindrical fire-clay furnace four and one-half inches in diameter, set in a wrought-iron case and covered by a clay dome, the whole being about six inches high. To the bottom of the iron case was attached a burner, by which a mixture of gas and air was carried to the centre of the furnace, where it was ignited. At the lower end of this burner tube entered two small tubes, through one of which gas was supplied, the other being used to conduct an air-blast from the bellows. On the clay bottom of the furnace inside are three little supports for the clay planche, or tray, on which the teeth are placed for the baking. This planche and the teeth are protected from the flame and blast by a small clay cover. The teeth could not be laid directly upon a planche, for they would stick to it when fused, so they are put upon some coarse clean silex. With this furnace the time of baking was reduced to twelve minutes, and the dirt problem was entirely eliminated.

The air blast was maintained by means of a bellows worked by the foot, or connected to a one-fourth horse-power motor and operated by electricity. This form of furnace I have used constantly with success and profit until within a year, when I adopted the Hammond furnace with the No. 2 muffle. As this furnace is well known to all of you, I will not describe it except to say that the time required to bake the porcelain that I use requires ten or twelve minutes to heat and seventeen minutes to bake; in all, I count on about one-half hour. My preference for an electric furnace is on account of the fact that I can have it in my operating-room, and, being noiseless and odorless, I can have baking going on while attending to a patient in the chair. I am also saved the trouble of going up and down two flights of stairs to my laboratory. Its capacity is somewhat limited, which is an objection. Were I in my laboratory nearly all the time, I would still prefer the gas furnace.

BAKING.

Baking with the gas furnace, when the tooth is thoroughly dried, put the tray containing it upon the supports in the bottom of the furnace, cover with the little clay cover, turn on the gas, light it, and then turn on the air-blast (either foot bellows or mechanical pump). Gradually increase the flow of both gas and air, and when both are on full force put the top of the furnace in position and continue the baking from ten to fourteen minutes. Shut off gas and air together and allow to cool gradually. If you wish a rapid cooling, take the furnace top off and remove tray and cover together, with appropriate tongs, and place in a large fire-clay muffle. Porcelain can be cooled comparatively quickly without accident, providing it is cooled equally and evenly. If, however, the top part cools more rapidly than the part which is in contact with the silex on the bottom, and which of course holds the heat longer, you will be sure to find your work cracked. I would never try to cool a thin gum section rapidly. With a gas furnace you must carefully regulate the gas supply at the stop-cock so that the flame visible at the orifice at the top of furnace is straw-color, thus showing perfect combustion and preventing "gassing." A blue flame will "gas" the baking. The baking may be examined by removing the top, but after a little experience one can bake by "time."

With the Hammond electric furnace No. 2 I use a little platinum planche and cover (gauge 34) on which to put my work. Start with cold furnace, heat ten minutes on first stop, two minutes each on the second, third, fourth, and fifth stops, and about fifteen minutes on the sixth stop. Total, thirty-three minutes from start to finish. Cool slowly, and do not remove the teeth from the furnace until it has cooled so that you can bear your hand upon the outside of the muffle dome.

In concluding this brief sketch of the history and art of carving teeth, I wish to say that I consider that the advantages of this sort of work over the ready-made teeth are, better colors, a more natural and lifelike appearance, the possibility of taking conditions in the mouth exactly as you find them, especially concerning crowns and partial dentures, the fact that you can put the pins exactly where needed for greatest strength, and lastly, a result which enables art to conceal art, pleases your patient, and satisfies your own inner consciousness.

FORMULAS FOR BODIES AND ENAMELS.

Thomas H. Chandler's body:

Silex, 1 oz.;
Felspar, 2 oz.;
Kaolin, 2 dwts.;
Yellow frit, 3 grs. and upward.

YELLOW FRIT.

Spar, 45 dwts.;
Ox. titanium, 5 dwts.;
Ox. chromium, 3 to 10 grs.

Drs. C. P. Wilson and G. T. Moffatt say that the old Harwood and Tucker body was:

Spar, 12 dwts.;
Silex, 8 dwts.;
Clay, 20 grs.;
Color frit.

Dr. A. H. Parker says that the above should be:

Spar, 4 oz.;
Silex, 3 oz.;
Clay, 1 dwt.; which is what he uses, and as he learned tooth carving of Dr. Harwood, and was with him many years, it is to be presumed he would know. My father and Dr. Wilson's father, Dr. E. T. Wilson, were both with Dr. Tucker, and they obtained the former formula from him.

Dr. S. F. Ham's formula:

BODY.

Spar, 45 dwts.;
Silex, 15 dwts.;
Clay, 2 dwts.;
Color frit.

ENAMEL.

Spar, 10 parts;
Silex, 1 part;
Color frit.

YELLOW FRIT FOR BODY.

Spar, 40 dwts.;
Titanium, 5 dwts.;
Ox. chromium, 2 to 10 grs.

For dark bodies double the amount of chromium in the frit.

GRAY FRIT.

Felspar, 15 dwts.;
Ammoniated muriate of platina, 1 dwt.

Grind the above dry, then wet sufficiently to form into a cake, fuse on a tile, and plunge into cold water while red hot. Grind and float off.

Dr. John D. Dickinson uses for body:

Spar, 2 oz.;
Silex, 1 oz.;
Clay, 2 dwts.;
Coloring.

ENAMEL.

Spar, 18 parts;
Silex, 2 parts;
Coloring.

Dr. A. H. Parker's enamel is:

Spar, 6 parts;
Silex, 1 part;

BLUE FRIT FOR ENAMELS.

Spar, 15 dwts.;
Platina sponge, 1 dwt.

YELLOW FRIT.

Spar, 45 dwts.;
Titanium, 5 dwts.

BROWN FRIT.

Spar, 40 dwts.;
Titanium, 5 dwts.
Ox. chromium, 10 grs.

GRAYISH FRIT.

Spar, 10 dwts.;
Titanium, 2 dwts.;
Manganese, 12 grs.

BLUE BODY FRIT.

Felspar, 19 dwts.;
Oxide cobalt, 1 dwt.

## Figures and Tables

**Figure f1:**
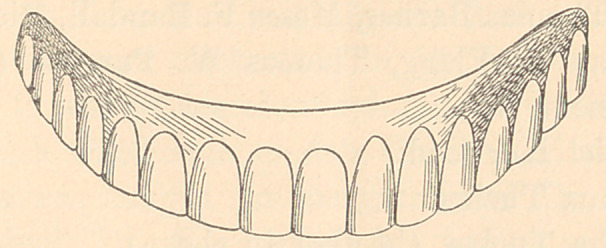


**Figure f2:**
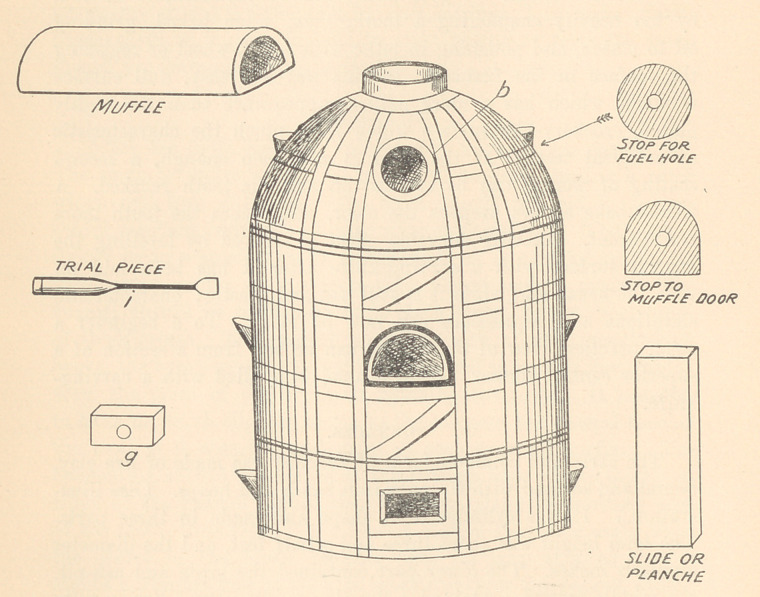


**Figure f3:**